# Effects of sub‐acute co‐exposure to WIFI (2.45 GHz) and *Pistacia lentiscus* oil treatment on wound healing by primary intention in male rabbits

**DOI:** 10.1002/vms3.753

**Published:** 2022-02-04

**Authors:** R. Latrach, N. Ben Chehida, A. Allous, H. Redid, A. Rejeb, H. Abdelmelek

**Affiliations:** ^1^ Surgery Service, Clinical Department National School of Veterinary Medicine of Sidi Thabet Sidi Thabet Tunisia; ^2^ Pathological Anatomy Service, Clinical Department National School of Veterinary Medicine of Sidi Thabet Sidi Thabet Tunisia; ^3^ Laboratory of Integrated Physiology, Faculty of Sciences of Bizerte University of Carthage Tunis Tunisia

**Keywords:** electromagnetic, high frequency, lentisk oil, skin, surgery, wound healing

## Abstract

**Background:**

The bioeffects of WIFI on cutaneous wound healing remains unexplored. In addition, several medicinal plant products including lentisk oil have been shown to interfere with wound healing process. Since the use of this oil is increasing, the co‐exposure (WIFI‐Lentisk oil) assessment is of paramount importance.

**Objectives:**

We aimed in the present study to investigate the effects of WIFI exposure as well as the application of *Pistacia lentiscus* oil on sutured wounds (SW).

**Methods:**

New Zealand male rabbits (*n* = 24) were used and randomly divided into four groups of six animals each: a control group (SW) and three experimental groups (i) a first group exposed to WIFI (2.45 GHz, 6 h/day) during 16 days (SWW); (ii) a second group exposed to WIFI (2.45 GHz, 6 h/day) during 16 days and treated with lentisk oil (SWWL) and (iii) a third group not exposed to WIFI but treated with lentisk oil (SWL). The wound healing was evaluated by monitoring clinical parameters (temperature, food intake, relative weight variation, and macroscopic aspect) and histology.

**Results:**

The mean food intake was higher in the SWWL group compared to the three other groups (*p* < 0.001) and higher in the SWL group compared to the SW group (*p* = 0.014). The exposition to WIFI (SWW group) or lentisk oil application (SWL group) can promote the collagen deposition and ameliorate the general aspect of wounds. By contrast, the co‐exposure to WIFI and lentisk oil (SWWL) results in antagonist effects and extends the inflammatory phase of wound healing.

**Conclusions:**

Wounds treated topically with *Pistacia lentiscus* oil should not be exposed to WIFI.

## Graphical abstract

In the present study, the bioeffects of co‐exposure to WIFI and *Pistacia lentiscus L*. oil on wound healing by primary intention in male rabbits were investigated. Used separately, they increased the collagen deposition and ameliorated the general aspect of wounds however the association extended the inflammatory phase of wound healing.

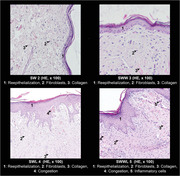

## 1 INTRODUCTION

Since the beginning of the 20th century, there has been a significant growth of processes and devices using electromagnetic fields (EMFs). They became indispensable in everyone's daily life (Saliev et al., 2014; Saunders, 2005). Such devices are being increasingly used in industry, engineering, telecommunications, education, home settings and medicine. In hospitals, different sources of EMFs are routinely used as WIFI, mobile phones and magnetic resonance imaging (MRI) devices. There are few studies exploring the bioeffects of EMFs on disease recovery especially on cutaneous wound healing (Rodemann et al., 1989; Saili et al., 2015) Previous investigations showed that pulsed EMFs improve early stages of wound healing and myofibroblast alignment in diabetic rats (Cheing et al., 2014; Goudarzi et al., 2010; Saliev et al., 2014). Choi et al. (2016) proved that the pulsed EMFs could enhance wound closure in the early healing stage. However, it seems to decrease the tensile strength of scar tissue during the remodelling phase. Few studies explored the bioeffects of ultra‐high‐frequency (UHF) EMFs as WIFI (2.45 GHz) on dermal wound healing. Latrach et al. (2018) proved that WIFI exposure increases collagen deposition and ameliorates the general aspect of skin sutured wounds in male rabbits.

The skin is the largest organ of the body that plays several roles among them the protection of the body against all external aggressions (Sorg et al., 2017). Many conditions can lead to inadequate wound healing which necessitates either medical or surgical treatments. Several teams are working on developing new approaches for skin wound healing, including the study of the potential effect of phytopharmacological methods (Poljšak et al., 2020; Vaughn et al., 2018). In dermatology, phytotherapy is the preferred option for patients over conventional therapy using chemical medicines, particularly in terms of side effects. Vegetable butters and oils have shown promising results in in vitro, in vivo and∕or clinical studies (Poljšak et al., 2020). In fact, the essential oils of plants can be an interesting strategy to prevent the bacterial growth and to promote the wound repair (Farahpour et al., 2020; Khezri et al., 2019). Many recent papers proved that *Cinnamon verum* essential oil accelerates wound healing process by increasing tissue antioxidant capacity and keratin biosynthesis in mice (Daemi et al., 2019; Seyed Ahmadi et al., 2019). The essential oil of *Mentha pulegium* and *Mentha piperita* promoted wound healing in mice by increasing antibacterial properties, decreasing inflammatory phase and accelerating the proliferation phase of wound healing process (Khezri et al., 2020; Modarresi et al., 2019). Recently, special emphasis was placed on Pistachio oil (*Pistacia lentiscus L.)* because it is one of the most widely used medicinal plants in the Mediterranean bassin including Tunisia (Belyagoubi‐Benhammou et al., 2018). Interestingly, lentisk oil exhibited many pharmacological effects such as antimicrobial, anti‐inflammatory, antidiabetic, antitumour and antioxidant activities (Abidi et al., 2017; Catalani et al., 2017; Ostovan et al., 2020). Given the interesting bioactivity of lentisk oil, we hypothesised that its topical application can accelerate the wound healing process. Accordingly, the aim of the present study was to investigate, for the first time as far as we know, the effects of lentisk oil on wound healing by primary intention in rabbits and to evaluate the effects of subacute WIFI (2.45 GHz) exposure on the quality of these wounds. The co‐exposure (WIFI‐Lentisk oil) had also been investigated.

## 2 MATERIAL AND METHODS

### 2.1 Ethical statement

The whole experiment was performed in accordance to the Tunisian code of practice for the care and use of animals for scientific purposes. The Tunisian Association of Laboratory Animals Science (ATSAL) approved the experimental protocol (No.0118 ATSAL).

### 2.2 Animals

The present study was carried out on 24 healthy New Zealand male rabbits obtained from the hutch of the National School of Veterinary Medicine of Sidi Thabet, Tunisia. These rabbits were of 3.60 ± 0.30 kg mean weight and aged of 1 year. All the animals were housed in standard environmental conditions of temperature (20 ± 3°C), humidity (60 ± 5%) under a 12:12 h light/ dark cycle with ad libitum access to water and commercial mash.

### 2.3 Surgery and clinical monitoring

Each rabbit was anesthetised by intramuscularly injection of 1 mg/kg body weight acepromazin and 20 mg/kg body weight Ketamin. The hair of each rabbit's lateral side of the thighs was shaved and aseptically prepared. In addition, cutaneous incision (3–4 cm length) was performed with a sterile blade (n°24). Moreover, surgical wound was sutured with simple interrupted stitches using synthetic braded absorbable suture fitted on triangular curved needle. The skin wound was disinfected with polyvinyl pyrolydone and aseptically covered with a sterile compress and an adhesive bandage. All the rabbits were monitored during surgery until awakening and daily, throughout the whole experimentation: they were clinically examined and their body temperature and food intake were measured daily. Rabbits were weighted before and after the experiment (days 1 and 16).

### 2.4 Exposure system

The rabbits were randomly divided into four groups of six animals each.

- SWW group: exposed to WIFI (2.45 GHz, 6 h/day) during 16 consecutive days.
- SWWL: exposed to WIFI (2.45 GHz, 6 h/day) during 16 days and treated with lentisk oil.
- SWL: not exposed to WIFI but treated with lentisk oil.
- SW: not treated with lentisk oil and not exposed to electromagnetic fields (EMFs).

### 2.5 Wound healing assessment

Wound healing was evaluated by monitoring both clinical and histological parameters. The healing process was monitored during 16 days throughout the experimental period to evaluate the wound healing. All photographs were double‐blinded evaluated for erythema, crusting/scabbing, pus formation and general wound appearance. These wound‐healing parameters were assessed using the three‐point scales for exploring irritation and infection (Bouaziz et al., 2014).

### 2.6 Histology

Skin biopsies (approximately 2 cm length and 1 cm large) including the lesion and the sound skin around the scar were taken, at day 16 from the animals of each group (Belyagoubi‐Benhammou et al., 2018; Pirbalouti et al., 2010).

The tissue samples were fixed in 10% formalin for 48 h, then processed by standard laboratory procedures. Briefly, they were embedded in paraffin to allow transverse sections of 3–5 μm thicknesses. Sections were stained with haematoxylin and eosin (HE) for histological examination by light microscopy.

Tissue samples were assessed for four criteria (epithelialisation, presence of inflammatory cells, presence of fibroblasts and collagen deposition response) using a four‐rating scale evaluation (from 0 to 3) (Lemo et al., 2010).

### 2.7 Statistical analyses

Quantitative results were reported as means ± SEM (standard error of mean). The difference of means between the four groups was compared by ANOVA test for food intake and body temperature. Body weight relative variations were compared, between day 1 and day 16, by Mann–Whitney test for each group and by Kruskal–Wallis test, at day 1 then at day 16, between the four groups at a threshold value of 5% (Schmitt et al., 1993). The normality was checked by the Kolmogorov–Smirnov test using SPSS® 26 for Windows®.

## 3 RESULTS

### 3.1 Effects of WIFI signals and lentisk oil application on food intake

**FIGURE 1:**
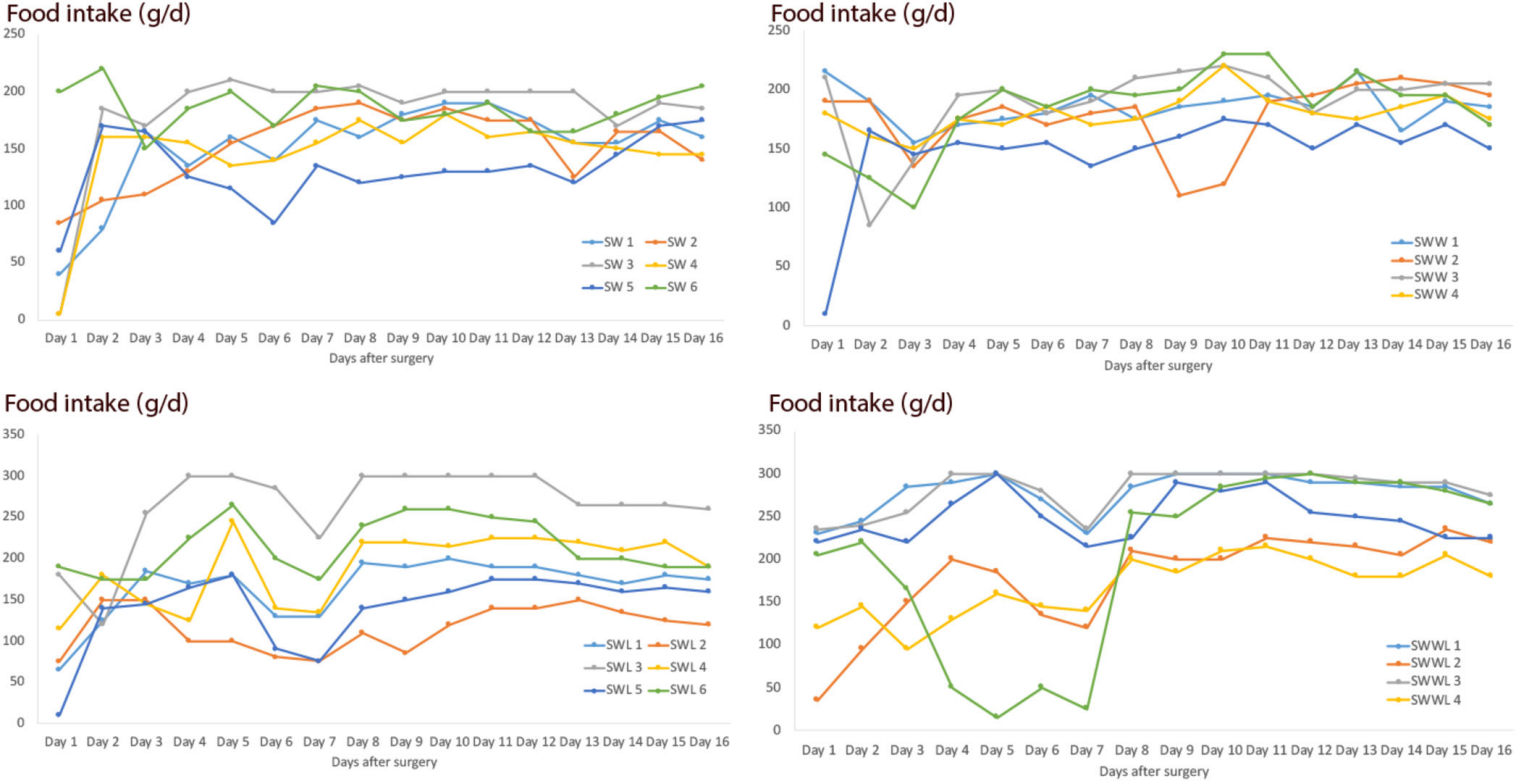
Food intake in rabbits in control group (SW), WIFI exposed (SWW), lentisk treated (SWL) and combination of WIFI and lentisk oil (SWWL). SW: rabbits with sutured wounds; SWW: rabbits with sutured wounds exposed to WIFI; SWL: rabbits treated topically with lentisk oil; SWWL: rabbits exposed to WIFI and treated with lentisk oil

The mean food intake was higher in the SWWL group compared to the three other groups (SW, SWW and SWL) (*p* < 0.001). Moreover, the food intake in the SWL group was higher than the SW group (*p* = 0.014) (Figure 1).

### 3.2 Study of co‐exposure to WIFI and lentisk oil on body weight

At day 1, the body weight of the four rabbit groups was comparable (*p* > 0.05).

For each group there was no difference of body weight relative variation between day 1 and day 16. Moreover, there was no difference between the four groups at both day 1 and day 16 (Table 1).

**TABLE 1:** Mean body weight of rabbits in control group (SW), WIFI exposed (SWW), lentisk oil treated (SWL) and combination of WIFI and lentisk oil (SWWL)

Rabbit groups	Body weight at day 1 (mean in kg ± SEM)	Body weight at day 16 (mean in kg ± SEM)	Relative variation (%)
SW	3.89 ± 0.40	3.77 ± 0.09	−0.02
SWL	3.54 ± 0.33	3.65 ± 0.06	0.03
SWW	3.37 ± 0.28	3.47 ± 0.03	0.02
SWWL	3.60 ± 0.38	3.85 ± 0.03	0.07

### 3.3 Study of co‐exposure to WIFI and lentisk oil on body temperature

**FIGURE 2:**
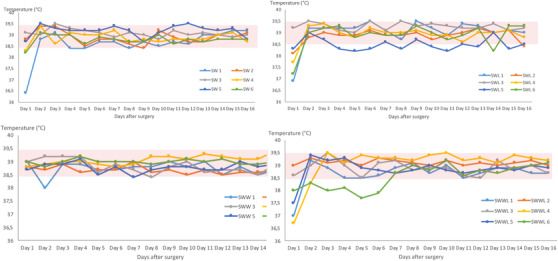
Thermographs of rabbits in control group (SW), WIFI exposed (SWW), lentisk treated (SWL) and combination of WIFI and lentisk oil (SWWL). SW: rabbits with sutured wounds; SWW: rabbits with sutured wounds exposed to WIFI; SWL: rabbits treated topically with lentisk oil; SWWL: rabbits exposed to WIFI and treated with lentisk oil

Co‐exposure to WIFI and lentisk oil have no effects on body temperature in four animal groups during the whole experiment (*p* < 0.05) (Figure 2).

### 3.4 Evaluation of macroscopic and microscopic wound's assessment in male rabbits

**FIGURE 3:**
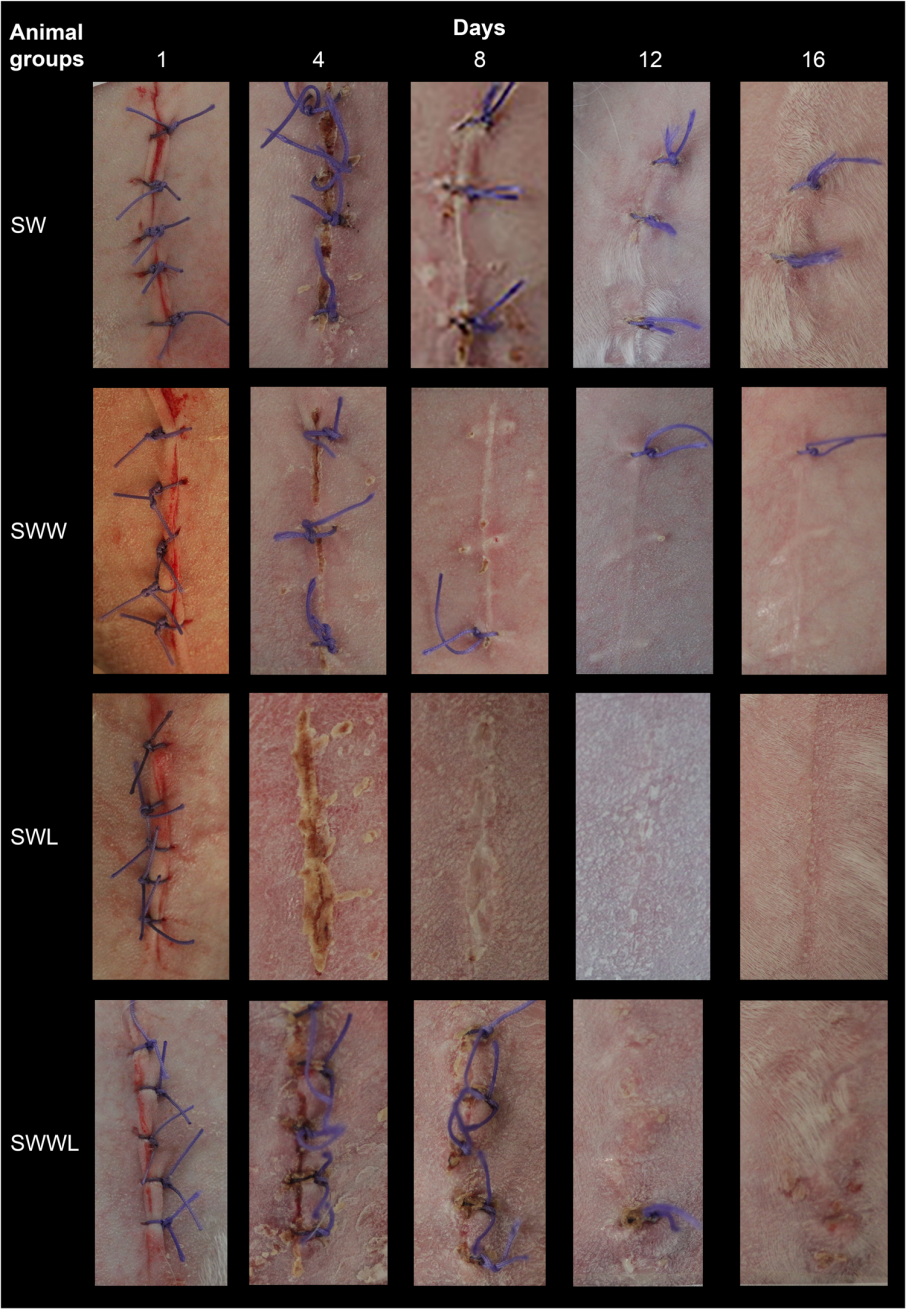
Representative photographs ds of four rabbit's wounds (SW, SWW, SWL and SWWL) at days 1, 4, 8, 12 and 16. SW: rabbits with sutured wounds; SWW: rabbits with sutured wounds exposed to WIFI; SWL: rabbits treated topically with lentisk oil; SWWL: rabbits exposed to WIFI and treated with lentisk oil

The studied groups wound's appearances are illustrated in Figure 3, showing the wounds aspect evolution from days 1, 4, 8, 12 until day 16 post‐surgery.

**FIGURE 4:**
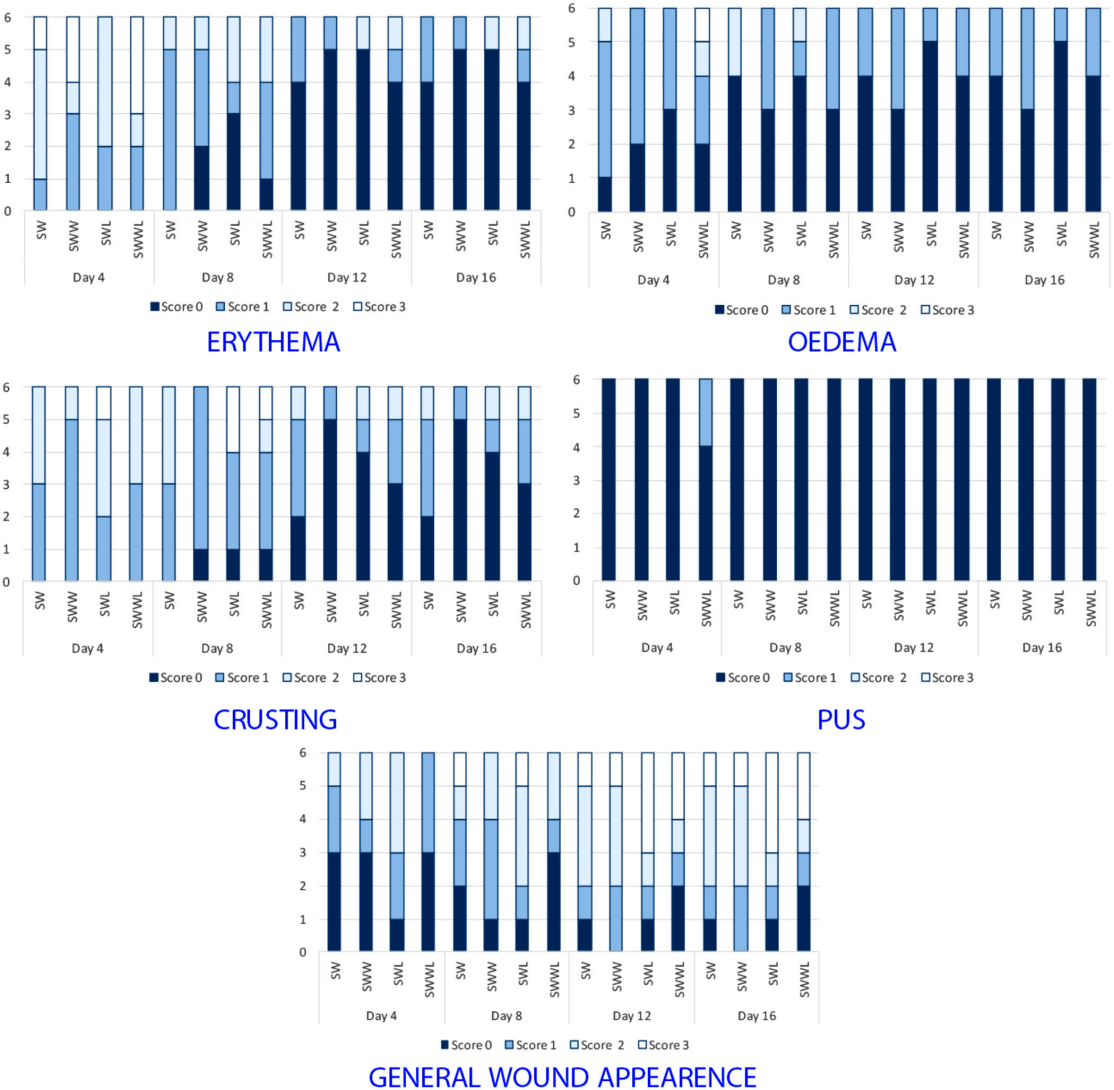
Parameters of macroscopic grading scales of the wounds for the four studied rabbit groups (SW, SWW, SWL and SWWL) on days 4, 8, 12 and 16. HE: haematoxylin and eosin; SW: rabbits with sutured wounds; SWW: rabbits with sutured wounds exposed to WIFI; SWL: rabbits treated topically with lentisk oil; SWWL: rabbits exposed to WIFI and treated with lentisk oil

At day 1, all wounds had similar bright red colour but from day 4, a brown red clot covering the wounds appeared in all rabbit groups, indicating the beginning of the wound healing process (Figure 4).

At day 4, the wounds were covered with scabs in the four rabbit groups that began to disappear on day 8 in SW, SWL and SWW groups, but persisted in SWWL group until day 16 (Figure 4).

The wounds in the control group (SW) and the SWW and SWL groups were relatively clean and did not develop any inflammatory reaction (erythema, swelling and redness). Moreover, at day 4, pus was surrounding the wounds of the SWWL group rabbits but at day 8, these wounds became more homogeneous and more consistent in texture (Figure 4).

Between day 4 and 8, erythema and oedema were observed in the four rabbit groups. Afterwards, SWL and SWW groups showed lower scores of erythema and oedema compared to the control group (SW). On the other hand, scores of erythema and oedema in the SWWL group were higher than both experimental groups (SWW and SWL) and control group SW (Figure 4).

At day 16, the wounds had good general appearance in the four groups (Figure 4) but the better results were obtained in the WIFI exposed group (SWW), the group treated with lentisk oil (SWL) and the control group (SW). In these groups, the skin surface was smooth and the colour was similar to normal skin. In contrast, the wounds of the SWWL rabbits were less homogenous and wounds repair was delayed comparison with the other groups.

**FIGURE 5:**
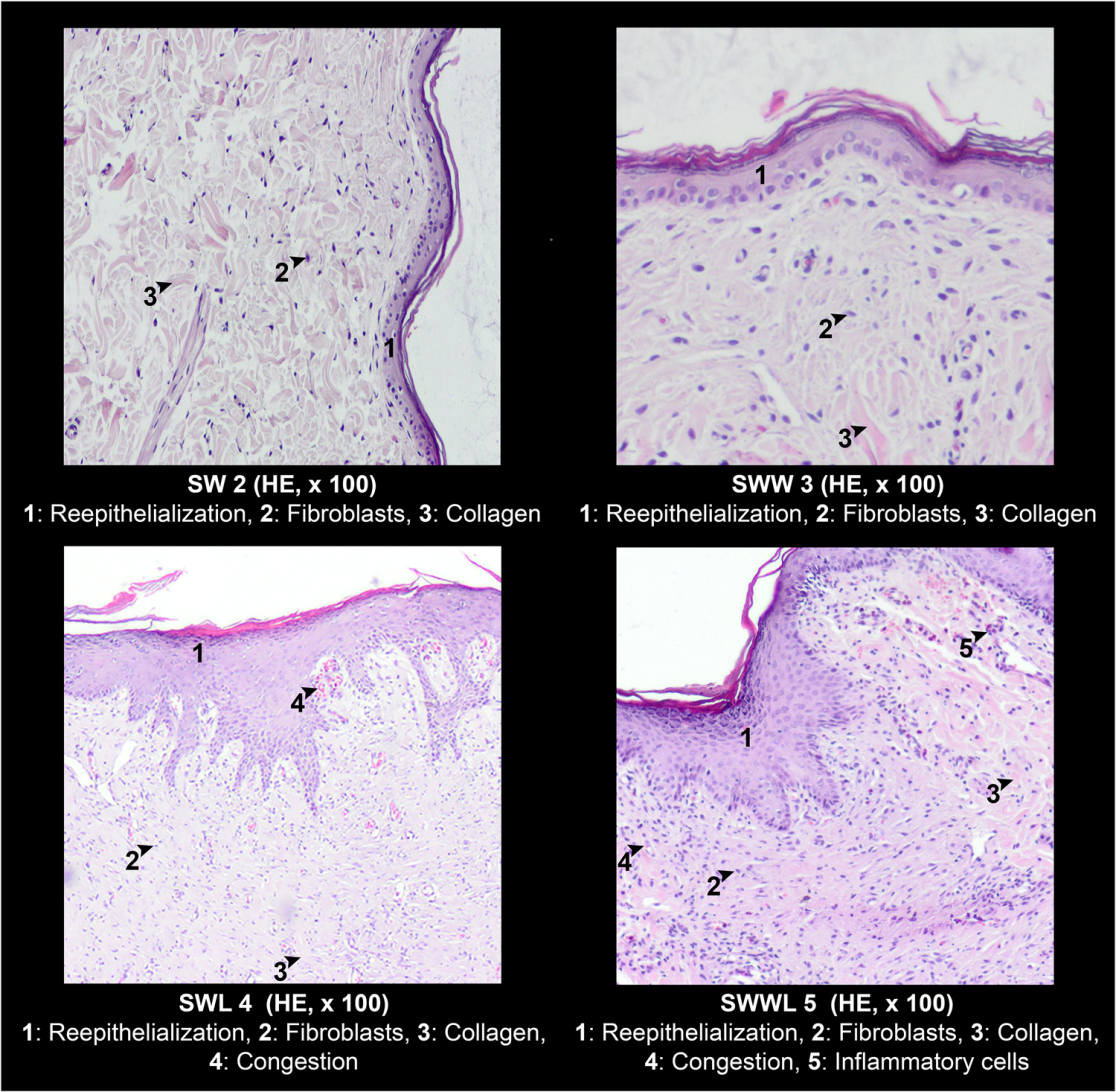
Micrographs of representative skin sections from rabbits’ skin wounds at day 16. SW: rabbits with sutured wounds; SWW: rabbits with sutured wounds exposed to WIFI; SWL: rabbits treated topically with lentisk oil; SWWL: rabbits exposed to WIFI and treated with lentisk oil

**FIGURE 6:**
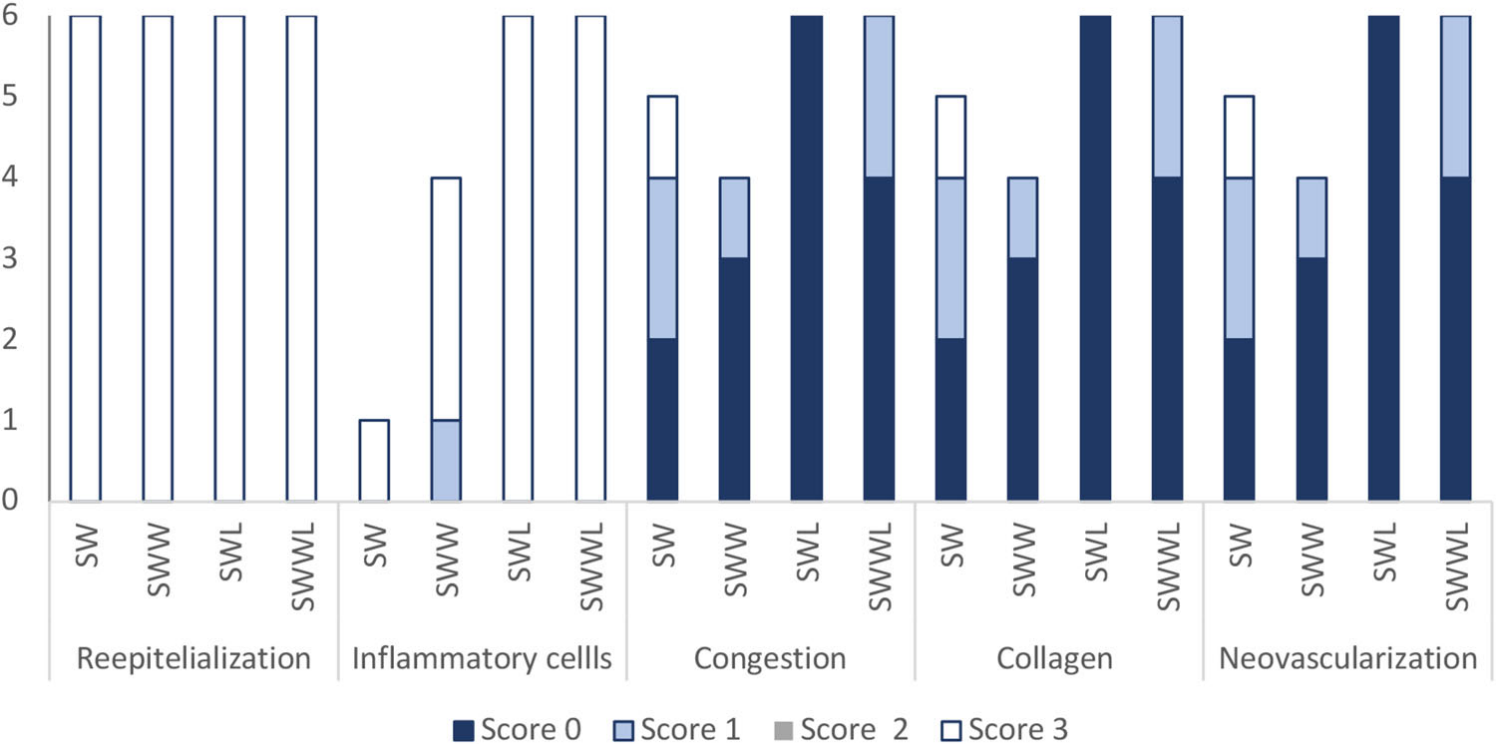
Parameters of histologic grading scales of the wounds for the four studied rabbit groups (SW, SWW, SWL and SWWL) on day 16. SW: rabbits with sutured wounds; SWW: rabbits with sutured wounds exposed to WIFI; SWL: rabbits treated topically with lentisk oil; SWWL: rabbits exposed to WIFI and treated with lentisk oil

Microphotographs taken during the histological assessment of the wound tissues at day 16 are presented in Figure 5 (graphical table content). The histological semi‐quantitative assessment of wound sections based on the 3‐point scale score showed complete wound re‐epithelisation the four groups. Interestingly, a higher number of fibroblasts was observed in the three experimental groups comparatively with the control group (Figure 6). In addition, inflammatory cells were noted in all histological samples but their number was more important in the SWWL group. In contrast, the collagen deposition was higher in the SWW and SWL groups (Figure 6).

## 4 DISCUSSION

The rapid development of technologies using EMFs and smart cities will certainly influence the human civilisations, animal behaviour and ecosystems and will probably interfere with xenobiotics and drug efficacies as previously demonstrated by Saili et al. (2015). These authors showed that WIFI (2.45 GHz) induce an increase in heart rate and arterial blood pressure of rabbits. In addition, Yorgancilar et al. (2017) investigated the long‐term effects of radiofrequency radiation emitted from WIFI systems on hearing in rats and proved that EMFs can significantly affect the hearing in adult Wistar rats. Recently, Vafaei et al. (2020) demonstrated that WIFI increased oxidative stress and apoptosis in mice's placenta tissue. By contrast, Tatarov et al. (2011) showed that exposure of nude mice, transplanted with breast tumoural cells, to magnetic fields for 360 min during 4 weeks suppressed tumour growth. In addition, Beck‐Broichsitter et al. (2014) suggested that the pulsed magnetic field therapy has a positive influence on neural regeneration in rats. However, the question of whether WIFI can influence skin healing remains not explored.

Skin is the mammalian body's largest organ that plays several vital roles. It constitutes an anatomical and physical interface between the internal and the external environments.

Topical application of vegetable butters and oils is a widely used traditional therapy (Poljšak et al., 2020). It continues to show promising results in the treatment of skin wounds as they have an effective impact on the wound‐healing phases. The observed biological effects could be explained in part by different processes through their antimicrobial, anti‐inflammatory and antioxidative activities (Poljšak et al., 2020; Serifi et al., 2019). In addition, vegetable butters and oils can promote cell proliferation, increase collagen synthesis, stimulate dermal reconstruction and repair the skin's lipid barrier function. Pistachio (*Pistacia lentiscus L.)* is one of the most widely used medicinal plants in the Mediterranean area including Tunisia (Belyagoubi‐Benhammou et al., 2018). This species contains many bioactive molecules, especially high concentration of phenolic compounds and fatty acids (Mezni et al., 2015). Indeed, pistachio extracts and oil exhibit many beneficial health effects such as antimicrobial, anti‐inflammatory, antidiabetic antitumour and antioxidant activities (Abidi et al., 2017; Catalani et al., 2017; Ostovan et al., 2020).

Taking into consideration animals’ welfare (3Rs: reduction, refinement and replacement), we reduced the number of animals in the present study. In each group, six rabbits were used (Fujita et al., 2017; Kim et al., 2020).

The choice of an affordable and reproducible experimental wound model is a cornerstone for objective investigations of the effects of different external factors such as WIFI exposure and/or vegetable oils application on skin wound healing. In general, there are two main models to investigate wound healing, that is, incisional and excisional allowing determination of the three phases of wound repair: inflammation, proliferation and maturation (Dorsett‐Martin & Wysocki, 2008).

For the excisional model, the wound closure is facilitated by centripetal contraction, but for incisional wounds with primary closure (sutured wounds), the edges of the wound are already stuck and contraction could not be measured. The excisional model is more appropriate for histological evaluation due to broader morphological changes occurring during the healing process whereas the incisional (sutured) skin healing model is preferred for wound tensile strength measurement (Davidson, 1998; Gàl et al., 2008). In our study, we used the sutured wounds model since it is widely employed in surgery.

As far as we know, the present investigation is the first work that evaluates the effects of co‐exposure to WIFI and lentisk oil on sutured wounds repair.

The study of Koyama et al. (2014) pointed that WIFI (2.45 GHz) modulates differentiated HL‐60 human cells during 4 and 24 h. In our study, we exposed the rabbits during 16 days to WIFI (2.45 GHz) in order to constitute a thick cell layer and to cover the three phases: inflammatory, proliferative and part of the remodelling phase of incisional wound healing process (Diegelmann & Evans, 2004).

Our results showed that the mean food intake was higher in the SWWL group compared to the three other groups (SW, SWW and SWL) (*p* < 0.001). Moreover, the food intake in the SWL group was higher than in the SW group (*p* = 0.014). This difference was probably related to the flavours of lentisk oil which gives a strong and characteristic smell stimulating appetite. Interestingly, WIFI exposure and treatment with *Pistacia lentiscus L*. oil have effects on neither the relative weight variation nor on the body temperature, for both control and experimental groups during the 16 days post‐surgery. These results are in accordance with the findings of Kim et al. (2015); they pointed that EMFs range from 10 MHz to 300 GHz do not have any thermal effect on organisms. In addition, Khedir et al. (2017) proved that lentisk oil do not affect the body weight in rats with induced laser burns.

On the basis of the wound photos taken on days 1, 4, 8, 12 and 16, we can conclude that WIFI exposure promoted early stages of wound healing until day 16. In addition, the application of lentisk oil improved the general wound appearance in treated rabbits. In fact, the general wound appearance was fair to good at day 4 in the SW, SWW and SWL groups and continually improves until day 16. These findings are in accordance with the study carried out by Latrach et al. (2018) who suggested that WIFI can promote the general wound appearance in rabbits exposed 6 h∕day to UHF EMFs (2.45 GHz). Moreover, Khedir et al. (2017) demonstrated high general wound appearance scores in the *Pistacia lentiscus L*. oil‐treated group compared with the other groups of rats with laser burns. In contrast, our data reveal a poor general wound appearance was poor following 8 days in the SWWL group.

Between day 4 and 8, erythema and oedema were observed in the four rabbit groups. From the 8th day, SWL and SWW groups showed lower scores of erythema and oedema compared to the control group (SW). On the other hand, scores of erythema and oedema in the SWWL group were higher.

At day 4, the wounds were covered with scabs in the four rabbit groups that began to disappear on day 8 in SW, SWL and SWW groups, but persisted in SWWL group until day 16. Latrach et al. (2018) reported less crusting during the experiment in the group exposed to WIFI (SWW) compared to the control group (SW) of male rabbits. In addition, Khedir et al. (2017) showed less crusting in the group treated with lentisk oil compared to the other groups.

In the same context, only two rabbits from SWWL group presented infected wound at day 4 but the pus shrunk and dried up at day 8. In contrast, the other groups presented no infection during all the experimental period. These results can be attributed to the antibacterial properties of the lentisk oil as demonstrated by Koutsoudaki et al. (2005) and WIFI (Latrach et al., 2018).

Our results showed that, in the SWWL group, the general wound appearance was poor, there was a persistence of oedema, erythema and scabs and there was suppuration in two rabbits’ wounds. Consequently, the co‐exposure to WIFI and lentisk oil application can generate an antagonistic effect which alters the efficacy of EMFs on wound healing and decreases the percutaneous absorption of lentisk oil.

The histological semi‐quantitative assessment of wound sections based on the 3‐point scale score showed complete wound reepithelisation in all rabbit's groups. Interestingly, an important number of fibroblasts were observed in the three experimental groups comparatively with the control group. Inflammatory cells were present in all histological samples but their number was higher than in the SWWL group. In contrast, the collagen deposition was higher in the SWW and SWL groups compared to SW and SWWL groups. In fact, it has been reported that pulsed EMFs stimulate fibroblasts and endothelial cells (Rodemann et al., 1989). In addition, amelioration of wound healing could be explained by increased collagen synthesis and angiogenesis. Farndale and Murray (1985) and Kim et al. (2015) demonstrated that EMFs is not cytotoxic and does not affect cell proliferation but it decreases in vitro wound healing by inhibiting cell migration.

Khedir et al. (2017) reported, at day 8, in the lentisk oil‐treated group, the presence of connective tissue characterised by a good collagen production. In addition, a study performed on rabbits with laser dermal burns proved that lentisk oil reduces the inflammatory phase, stimulates wound contraction and reduces the epithelialisation period compared to the two control groups treated with Vaseline or a cream for dermal healing and ulcers treatment (Madecassol^®^) (Djerrou et al., 2013). In fact, a previous phytochemical analysis of Tunisian *Pistacia lentiscus L*. oil revealed the presence of a high concentration of polyunsaturated fatty acids (73.44%), mainly fatty oleic acid (45.66%) and linolenic acid (24.21%) (Mezni et al., 2012). It was also suggested that, in one hand, the fatty acids of the lentisk oil are able to reduce epidermal water loss and increase skin hydration (McGaw et al., 2002) which proves the important therapeutic implication of lentisk oil and a pertinent role on wound repair (Cardoso et al., 2004). In the other hand, linoleic and oleic acids have anti‐inflammatory properties that play a major role in the recruitment of inflammatory cells and accelerate the wound healing process. Indeed, linoleic acid is a precursor of arachidonic acid in wounds. Arachidonic acid‐generated inflammatory mediators that can improve local neovascularisation are extracellular matrix reorganisation, cellular migration and fibroblastic differentiation. As a matter of fact, the linoleic acid that was identified in the lentisk oil accelerates the epidermis differentiation and the wound repair. The antioxidant effect of lentisk oil may also promote wound healing (Fitzmaurice et al., 2011). The quantification of total tocopherol in lentisk oil showed that a‐tocopherol is the major tocopherol fraction. Several previous studies demonstrated that a‐tocopherol oral supplementation promotes the healing of chronic wounds (Mezni et al., 2014). The present investigation reported for the first time, as far as we know, a modulatory effect of WIFI and∕ or lentisk oil on sutured wounds in male rabbits.

## 5 CONCLUSION

Our study reported that WIFI (2.45 GHz) exposure increases the collagen deposition and ameliorates the general aspect of wounds in male rabbits. Pistachio oil (*Pistacia lentiscus L*.) application on wounds ameliorates also the general wound appearance and the rate of collagen. However, the co‐exposure to WIFI (2.45 GHz) and *Pistacia lentiscus L*. oil extends the inflammatory phase of wound healing. Consequently, the topical application of lentisk oil on sutured wounds is recommended and would be an economically interesting alternative to synthetic products. However, it can be suggested that wounds treated topically with *Pistacia lentiscus L*. oil in humans and animals should not be exposed to WIFI. More studies are needed to investigate the bioeffects of WIFI and lentisk oil on several other wound models (excisional wounds, burns and infected wounds).

## CONFLICT OF INTEREST

The authors declare that there are no conflicts of interest.

## AUTHOR CONTRIBUTIONS

**Rym Latrach**: conceptualisation, data curation, formal analysis, investigation, methodology, writing‐original draft and writing‐review & editing. **Noureddine Ben Chehida**: methodology, writing‐original draft and conceptualisation. **Aymen Allous**: methodology and writing‐original draft. **Helmi Redid**: methodology and writing‐original draft. **Ahmed Rejeb**: conceptualisation and methodology. **Hafedh Abdelmelek**: conceptualisation, supervision and writing‐review & editing.

## ETHICS STATEMENT

The whole experiment was performed in accordance to the Tunisian code of practice for the care and use of animals for scientific purposes. The Tunisian Association of Laboratory Animals Science (ATSAL) approved the experimental protocol (No. 0118 ATSAL).

## FUNDING

This study was resulted from a thesis research project and supported by Author's own work.

### PEER REVIEW

The peer review history for this article is available at https://publons.com/publon/10.1002/vms3.753

## Data Availability

Data sharing not applicable to this article as no datasets were generated or analysed during the current study.
